# Dulaglutide Protects Mice against Diabetic Sarcopenia-Mediated Muscle Injury by Inhibiting Inflammation and Regulating the Differentiation of Myoblasts

**DOI:** 10.1155/2023/9926462

**Published:** 2023-08-07

**Authors:** Fengyi Deng, Wenyan Wu, Xingyu Fan, Xing Zhong, Nuojin Wang, Yue Wang, Tianrong Pan, Yijun Du

**Affiliations:** ^1^Department of Endocrinology, The Second Affiliated Hospital of Anhui Medical University, Hefei 230061, China; ^2^Research Center for Translational Medicine, The Second Affiliated Hospital of Anhui Medical University, Hefei 230061, China

## Abstract

**Background:**

Type 2 diabetes mellitus increases the risk of sarcopenia, which is characterized by decreased muscle mass, strength, and function. However, there are no effective drugs to treat diabetic sarcopenia, and its underlying mechanism remains unknown. Here, we aimed to determine whether the GLP-1 receptor agonist (GLP-1RA) dulaglutide (Dul) affects the progression of diabetic sarcopenia.

**Methods:**

db/db mice were injected intraperitoneally with 0.6 mg/kg dulaglutide for 10 weeks. Mouse muscle tissues were then pathologically evaluated and stained with F4/80 or MPO to detect macrophages and neutrophils, respectively. In addition, inflammatory factors and FNDC5 in the muscle tissues were detected using qRT-PCR. Moreover, C2C12 cells were induced to enable their differentiation into skeletal muscle cells, and muscle factor levels were then detected. Furthermore, changes in muscle factor levels were detected at various glucose concentrations (11 mM, 22 mM, and 44 mM).

**Results:**

In vivo, dulaglutide alleviated muscle tissue injury; reduced levels of the inflammatory factors, IL-1*β*, IL-6, CCL2, and CXCL1; and reversed the level of FNDC5 in the muscle tissues of db/db mice. In vitro, a C2C12 cell differentiation model was established through the observation of cell morphology and determination of myokine levels. Upon stimulation with high glucose, the differentiation of C2C12 cells was inhibited. Dulaglutide improved this inhibitory state by upregulating the levels of both FNDC5 mRNA and protein.

**Conclusions:**

Treatment with the GLP-1RA dulaglutide protects db/db mice against skeletal muscle injury by inhibiting inflammation and regulating the differentiation of myoblasts. High glucose inhibited the differentiation of C2C12 cells and decreased the mRNA and protein levels of myokines. Dulaglutide could reverse the differentiation state induced in C2C12 cells by high glucose.

## 1. Introduction

The prevalence of diabetes mellitus is gradually increasing. The numbers of individuals suffering from diabetes in China and India were 145 million and 74 million, respectively, in 2021, accounting for 41% of the global adult diabetic population [1]. Sarcopenia is a muscle-wasting syndrome characterized by the progressive and systemic degenerative loss of skeletal muscle mass and strength [2, 3]. Both aging and Type 2 diabetes mellitus (T2DM) are risk factors for sarcopenia, and the risk of sarcopenia is significantly increased in elderly patients with T2DM. Studies have shown that the prevalence of sarcopenia in elderly patients with T2DM is 2-3 times higher than that in the average population and can reduce physical activity and daily activities, seriously affecting the quality of life and increase the hospitalization rate, medical expenses, and risk of death in the elderly [4–6]. Therefore, new therapeutic strategies that can improve T2DM with sarcopenia are awaited.

Diabetes and sarcopenia affect each other. Glucose metabolism disorders promote catabolism and muscle protein decomposition, leading to decreased muscle function and muscle content [7]. The decrease in muscle content further aggravates insulin resistance in muscles, which inhibits energy metabolism in the mitochondria of muscle cells and affects the normal contractile function of muscle tissue [8]. Both myokines and inflammation play an essential role in the pathogenesis of T2DM and sarcopenia [9]. Various inflammatory cells release pro-inflammatory mediators in skeletal muscles. Subsequently, muscle cells are damaged or even die, resulting in the loss of muscle contractile properties [10]. The myokines secreted by skeletal muscles include myostatin, Fibronectin Type III Domain-containing Protein 5 (FNDC5), insulin-like growth factor 1 (IGF1), fibroblast growth factor 21 (FGF21), etc. [11]. At present, besides conventional lifestyle interventions, namely physical exercise and caloric control, calcium and vitamin D supplements, mesenchymal stem cell therapy, and myostatin inhibitors, are used for the treatment of diabetic sarcopenia [12–14]. However, none of these can improve sarcopenia to a great extent. Therefore, it is urgent to find new means to treat sarcopenia.

Glucagon‐like peptide‐1 receptor agonists (GLP‐1RAs) have been developed as an anti‐diabetic therapy to promote insulin secretion in T2DM [15]. A study reported that the activation of GLP-1R signaling may be helpful in the treatment of atrophy-related muscle diseases [16]. A recent study found that GLP-1RAs can recover muscle weakness by suppressing muscle inflammation and muscle fiber necroptosis [17]. We therefore hypothesized that GLP‐1RAs exert beneficial effects on T2DM with sarcopenia to recover muscle strength, suppress muscle inflammation, and regulate myokines. Dulaglutide, as a weekly preparation of GLP-1, has been shown to be beneficial in improving diabetes mellitus associated with chronic kidney disease as well as cardiovascular disease in clinical trials [18, 19]. Here, we show that dulaglutide protects against T2DM with sarcopenia by inhibiting inflammation and regulating the differentiation of myoblasts using *in vivo* and *in vitro* models of T2DM with sarcopenia.

## 2. Materials and Methods

### 2.1. Cell Culture and Treatment

C2C12 cells were cultured in Dulbecco's modified Eagle's medium (DMEM) containing 10% fetal bovine serum (FBS) and 1% penicillin-streptomycin (PS) at 37°C in a humidified atmosphere containing 5% CO_2_. In the exponential growth phase, the cells were inoculated into a 6-well plate (4 × 10^5^/well) and incubated at 37°C for 24 h. The culture medium was discarded at this point, and 2 mL of differentiation medium (containing 2% horse serum, 97% DMEM, and 1% penicillin-streptomycin) was added. After 4 days of culture, morphological changes in the C2C12 cells were observed using microscopy. Similarly, different concentrations of glucose (11 mM, 22 mM, and 44 mM) and 100 nM dulaglutide were added to differentiated cells, according to the assigned group treatment, and cells were collected 24 h later for further analysis.

### 2.2. Sulforhodamine B (SRB) Colorimetric Assay

C2C12 cells in the logarithmic phase were plated in 96-well plates containing 8 × 10^3^ cells per well. At the end of the drug action time, 50% TCA solution was added to each well to fix the cells, and the cells were then transferred to a refrigerator at 4°C for overnight incubation. After drying, 70 *μ*L of 0.4% SRB staining solution was added to each well. After staining for 30 min, the staining solution was poured out, and the cells were rinsed with 1% acetic acid five times and then dried at room temperature. The dye was dissolved in 100 *μ*L of non-buffered tris-base lye (10 mM, pH = 10.5), and the light absorption value in each well was measured at 540 nm using a microplate reader.

### 2.3. Animal Treatment

Specific pathogen-free (SPF) male diabetic db/db mice and nondiabetic littermate db/m mice (10 weeks) were purchased from Nanjing Mairuisi Biotechnology. All mice were kept in a controlled environment, allowed ad libitum access to food and water, and habituated to the room used to house them for 7 days before being subjected to experiments. After adaptation, the mice were divided randomly into four groups: a control group (db/m, 0.9% saline), a model group (db/db, 0.9% saline), a treatment group (db/db, dulaglutide 0.6 mg/kg), and a drug control group (db/m, dulaglutide 0.6 mg/kg), with six animals in each group. The Dul dosage selection was determined based on previous relevant experiments [20]. Drugs were administrated using intraperitoneal injection each week. For the experiment, one drop of mouse blood was collected using tail vein puncture, and the blood glucose level was measured weekly using a blood glucose meter. All the mice were euthanized 10 weeks after drug treatment. Blood samples were collected at the time of euthanasia. Muscle tissue was partly fixed in 4% phosphate-buffered formaldehyde for histological analyses and partly snapped frozen for subsequent molecular analysis. All animal experiments were performed following the National Institutes of Health Guide for the Care and Use of Laboratory Animals, with the approval of the Center for scientific research at the Second Affiliated Hospital of Anhui Medical University (20211123).

### 2.4. Histologic Evaluation

Muscle sections (5 *μ*m) were stained with hematoxylin & eosin (H&E). Briefly, fresh muscle tissues were collected, fixed with 4% paraformaldehyde, embedded in paraffin, and sliced. The tissue slices were then stained with HE stain to observe histological changes in the muscles. Muscle injury was evaluated by a pathologist in a blinded manner and was assessed using the Manickam R method, as previously described [21]. Images were obtained using an Olympus BX41 microscope.

### 2.5. Immunohistochemistry

After deparaffinization and rehydration, 0.01 M citrate buffer (pH 6.0) was added to the tissue sections for antigen retrieval. Then, the sections were blocked with 10% goat serum for 30 min at room temperature. Next, the sections were incubated with F4/80 (Affinity, cat number: DF2789, 1 : 300) and MPO (Proteintech, cat number: 22225-1-AP, 1 : 400) antibodies overnight at 4°C. Subsequently, an HRP-DAB system (ZSGBBIO, Beijing, China) was used to detect immunoactivity in the tissue sections. This was followed by counterstaining with hematoxylin. After stepwise dehydration, the tissue sections were sealed, and their images were obtained using an Olympus BX41 microscope.

### 2.6. Quantitative Real-Time PCR

Total RNA was extracted from the treated muscle tissues using the TRIzol reagent (Thermo Fisher Scientific, Waltham, USA) and transformed to cDNA using the HyperScriptTM III 1st Stand cDNA Synthesis Kit (NovaBio, Shanghai, China). Quantitative real-time PCR was performed using the S6 Universal SYBR qPCR mix (NovaBio, Shanghai, China) and an ABI 7900 PCR system (ABI, USA). The 2^−ΔΔ*Ct*^ method was used to assess relative gene expression, and GAPDH was used for normalization of data. All primers were custom-made by Genscript. Primer sequences are listed in Supplementary Table 1.

### 2.7. Western Blotting Analysis

Briefly, protein samples were extracted from the lung tissues, boiled for 5 min, and electrophoresed in a 12% SDS polyacrylamide gel. They were then transferred onto PVDF membranes (Millipore, Billerica, MA, USA). The membranes were then blocked with 5% skimmed milk in Tris-buffered saline–Tween 0.1% for 2 h at room temperature, and then incubated with primary antibodies against FNDC5 (Abcam, cat number: ab174833, 1 : 1000), myostatin (Abcam, cat number: ab124721, 1 : 1000), PGC-1*α* (Abcam, cat number: ab176328, 1 : 1000), and GAPDH (Affinity, cat number: AF7021, 1 : 5000) at the appropriate dilutions overnight at 4°C. Next, the blots were washed and incubated for 1 h at room temperature with an HRP-conjugated secondary antibody, following which they were developed using enhanced chemiluminescence reagents (Affinity, Jiangsu, China). Densitometric analysis of protein bands was performed using the Image J software (National Institutes of Health, Bethesda, MD, USA).

### 2.8. Statistical Analysis

Data are expressed as mean ± SEM, and statistical analyses were performed using GraphPad Prism 8.0. Statistical significance of differences between two groups was analyzed using Student's *t*-test and that of differenced between more than two groups was analyzed using one-way ANOVA followed by Turkey's multiple comparison test. *P* < 0.05 was considered statistically significant.

## 3. Results

### 3.1. Dulaglutide Inhibited Diabetes-Induced Muscle Atrophy

**Figure 1:**
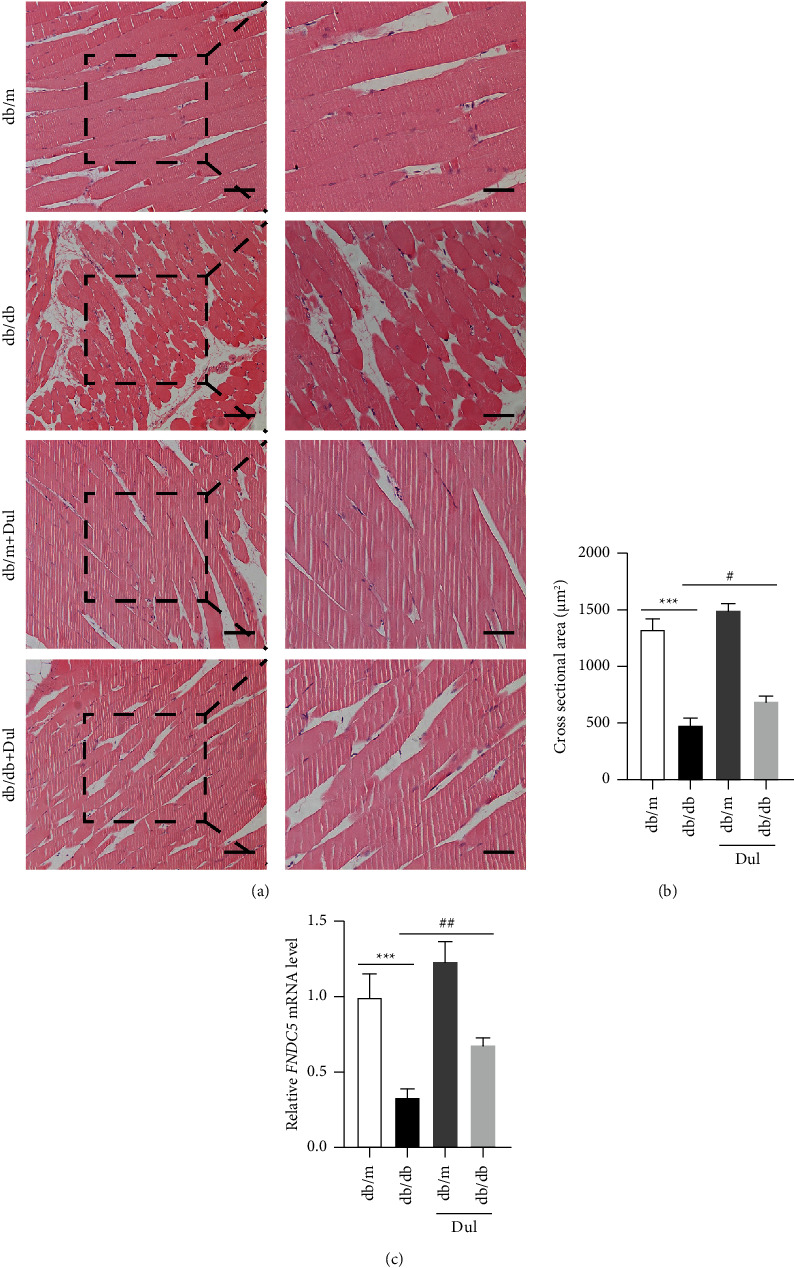
Dulaglutide treatment inhibited diabetes‐induced muscle atrophy. (a) Histopathological image of HE-stained muscles (scale bars: 100 *μ*m and 50 *μ*m). (b) Statistical analysis of the mean cross-sectional area of soleus muscles. (c) RNA was extracted from the soleus muscles, and the mRNA levels of FNDC5 were detected. ^*∗∗∗*^*P* < 0.001, ^*∗*^comparison between the db/db group and control db/m group; ^#^*P* < 0.05, ^##^*P* < 0.01, ^#^comparison between the dulaglutide-treated group and db/db group.

After being administered dulaglutide for 10 weeks, the db/db mice were dissected, and their soleus muscles were fixed, sectioned, and subjected to HE staining. Statistical analysis showed that the average cross-sectional area of db/db mouse soleus muscles was significantly decreased compared to that of age-matched db/m mouse soleus muscles, but this reduction in the cross-sectional area of db/db mouse soleus muscles could be inhibited by dulaglutide (Figures 1(a) and 1(b)). Contrarily, the mRNA level of FNDC5 in db/db mouse soleus muscles of was significantly decreased compared to that in db/m mouse soleus muscles; however, treatment with dulaglutide increased the expression of FNDC5 in the soleus muscles of both db/m and db/db mice (Figure 1(c)).

### 3.2. Dulaglutide Inhibited the Inflammatory Response in Diabetes-Induced Muscle Atrophy

**Figure 2:**
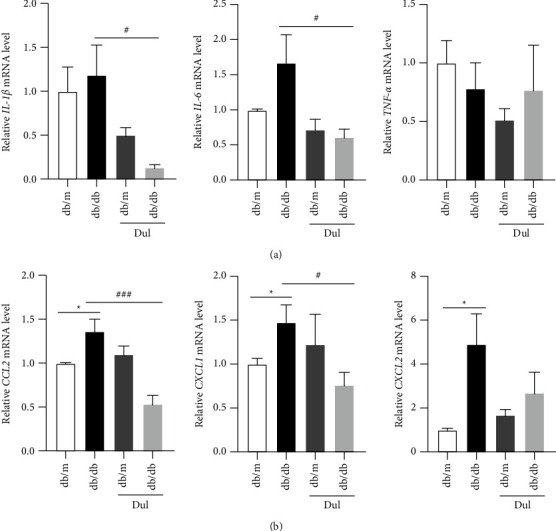
Dulaglutide inhibited the inflammatory response in mice with diabetes‐induced muscle atrophy. (a-b) The mRNA levels of IL-1*β*, IL-6, TNF-*α*, CCL2, CXCL1, and CXCL2 in soleus muscles were measured using qRT-PCR. ^*∗*^*P* < 0.05, ^*∗*^comparison between the db/db group and control db/m group; ^#^*P* < 0.05, ^###^*P* < 0.001, ^#^comparison between the dulaglutide-treated group and db/db group.

Inflammation is often involved in the pathogenesis of sarcopenia. Therefore, we used qRT-PCR to measure the expression of a series of inflammatory factors and chemokines. The mRNA levels of IL-1*β* and IL-6 in skeletal muscles were not notably different between db/m and db/db mice but were decreased upon dulaglutide administration in db/db mice (Figure 2(a)). In addition, the mRNA levels of CCL2, CXCL1, and CXCL2 in skeletal muscles were markedly increased in db/db mice compared to those in db/m mice. The mRNA levels of CCL2 and CXCL1 were significantly reduced in db/db mice upon treatment with dulaglutide, although the mRNA level of CXCL2 did not change significantly upon treatment with dulaglutide (Figure 2(b)).

### 3.3. Dulaglutide Attenuated Inflammatory Cell Infiltration in Diabetes-Induced Muscle Atrophy

**Figure 3:**
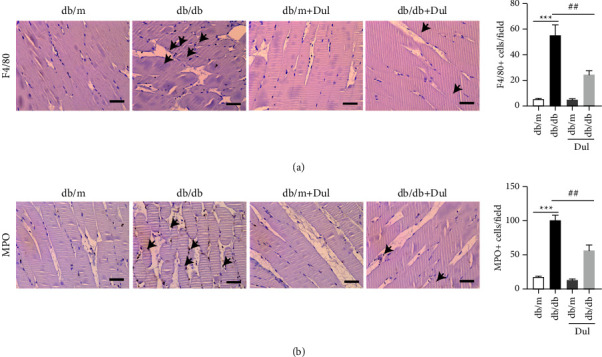
Dulaglutide attenuated inflammatory cell infiltration in diabetes‐induced muscle atrophy. (a, b) The macrophage marker F4/80 and neutrophil marker MPO in the soleus muscles of mice were stained and their expression levels were analyzed using immunohistochemistry. The scale bars represent 100 *μ*m and 50 *μ*m, respectively. ^*∗∗∗*^*P* < 0.001, ^*∗*^comparison between the db/db group and control db/m group; ^##^*P* < 0.05, ^#^comparison between the dulaglutide-treated group and db/db group.

In addition to changing the levels of inflammatory factors in the blood, chemokines also induce inflammatory cells to migrate and infiltrate into tissues and organs secreting them. Neutrophils are the earliest infiltrating inflammatory cells in acute inflammation, while macrophages are the primary functional cells involved in chronic inflammation. Our immunohistochemistry results showed that, compared with those in db/m mouse soleus muscles, the levels of the neutrophil marker MPO and macrophage marker F4/80 in db/db mouse soleus muscles were significantly increased; these levels were significantly decreased in db/db mice after they were treated with dulaglutide (Figures 3(a) and 3(b)).

### 3.4. Effect of Different Glucose Concentrations on the Proliferation and Differentiation of C2C12 Cells

**Figure 4:**
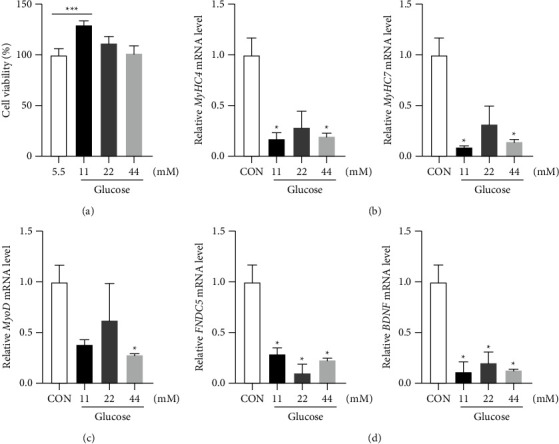
Effect of different glucose concentrations on the proliferation and differentiation of C2C12 cells. (a) C2C12 cells were plated in 96-well plates, and the SRB assay was used to detect the viability of C2C12 cells after administration of glucose at different concentrations. (b–d) C2C12 cells were treated with varying glucose concentrations, and the mRNA expression levels of the differentiation genes MyHC4 and MyHC7, the myogenic factor MyoD, and the muscle secretory factors FNDC5 and BDNF were detected. ^*∗∗∗*^*P* < 0.001, ^*∗*^comparison between the high glucose concentration group and the normal group.

C2C12 cells were stimulated with 2% horse serum to differentiate them into myotubes and increase the mRNA levels of the myocyte differentiation genes MyHC4 and MyHC7 and myokine factors MyoD and MyoG in them (Figures S1(a)–S1(c)). To analyze the effect of glucose on myocyte differentiation, differentiated mouse C2C12 myotubes were stimulated with different concentrations of glucose (11 mM, 22 mM, and 44 mM). The SRB assay was used to determine whether glucose had any effect on the viability of C2C12 cells. The results showed that high glucose promoted the proliferation of C2C12 cells, and cell proliferation was weakened with a further increase in the glucose concentration. The effect of 11 mM glucose on C2C12 cell proliferation was the most significant (Figure 4(a)). However, after stimulati n with a high glucose concentration, the mRNA levels of the differentiation-related gene MyHC4, MyHC7, the myogenic factor MyoD, and the muscle secretory factors FNDC5 and BDNF were decreased in differentiated C2C12 cells (Figures 4(b)–4(d)).

### 3.5. Expression of Myostatin, PGC-1*α*, and FNDC5 in C2C12 Cells Treated with Different Glucose Concentrations

**Figure 5:**
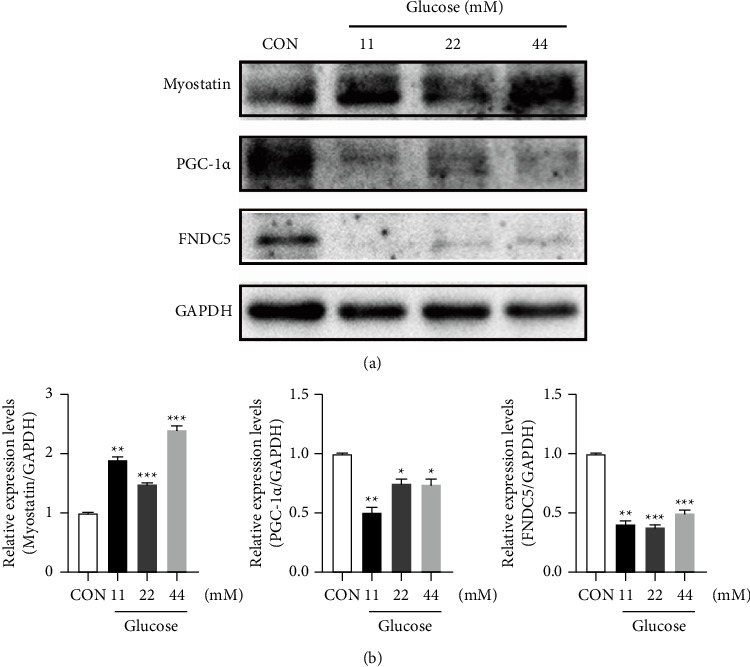
Expression of myostatin, PGC-1*α*, and FNDC5 in C2C12 cells treated with different glucose concentrations. (a) C2C12 cells were induced to differentiate and then treated with different glucose concentrations for 24 h. The C2C12 protein was extracted, and the expression levels of myostatin, FNDC5, and PGC-1*α* proteins were detected. (b) The Image J software was used to analyze the gray value of proteins after the stimulation of C2C12 cells with high glucose concentrations, and statistical analysis was performed. ^*∗*^*P* < 0.05, ^*∗∗*^*P* < 0.01, ^*∗∗∗*^*P* < 0.001, ^*∗*^comparison between the high glucose concentration group and the normal group.

C2C12 cells were differentiated and stimulated with glucose at different concentrations (11 mM, 22 mM, and 44 mM). Proteins were then extracted from C2C12 cells stimulated with varying concentrations of glucose (11 mM, 22 mM, and 44 mM). The expression level of the myostatin protein increased in response to high-glucose stimulation, while the expression levels of the FNDC5 and PGC1-*α* proteins decreased (Figures 5(a) and 5(b)). These results indicated that high glucose concentrations affected the expression levels of muscle factors in C2C12 cells.

### 3.6. Effect of Dulaglutide on C2C12 Cells Treated with High Concentrations of Glucose

**Figure 6:**
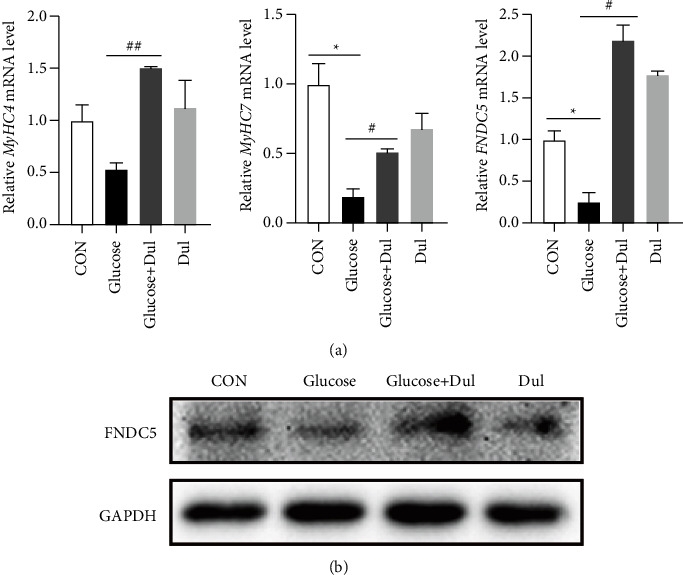
Effect of Dulaglutide on C2C12 cells treated with high glucose concentrations. (a) C2C12 cells were induced to differentiate and treated with 100 nM dulaglutide and 11 mM glucose for 24 h. RNA was extracted from these C2C12 cells, and the expression levels of MyHC4, MyHC4, and FNDC5 were detected. (b) Proteins were extracted from C2C12 cells, and the WB assay was performed for FNDC5. ^*∗*^*P* < 0.05, ^*∗*^comparison of CON between the glucose group and control group; ^#^*P* < 0.05, ^##^*P* < 0.01, ^#^comparison between the dulaglutide treated.

To investigate the effect of dulaglutide on C2C12 cells stimulated by high glucose, we administered 100 nM dulaglutide and 11 mM glucose to differentiated C2C12 cells simultaneously and measured mRNA and protein expression levels after 24 h of culture. The high glucose concentration increased the mRNA levels of MyHC4, MyHC7, and FNDC5 and the protein level of FNDC5 in differentiated C2C12 cells, while dulaglutide decreased these levels (Figures 6(a) and 6(b)). These findings indicated that dulaglutide could reverse the effects of high glucose concentrations on myocyte differentiation and the FNDC5 expression, suggesting that high glucose concentrations could affect the differentiation of C2C12 cells, and dulaglutide could improve myocyte differentiation and increase the effect of FNDC5.

## 4. Discussion

In the present study, we first found that dulaglutide improved pathological changes in muscles and inhibited inflammation related to diabetic sarcopenia in db/db mice. Moreover, we found that dulaglutide reversed the reduction in the FNDC5 mRNA level induced by high glucose concentrations in db/db mice. Next, we investigated whether the differentiation of C2C12 cells into skeletal muscle cells could be induced by high glucose concentrations. We found that dulaglutide could improve myocyte differentiation in C2C12 cells stimulated with high concentrations of glucose. These results suggest that dulaglutide may protect against diabetic sarcopenia by regulating inflammation and myokine levels.

Type 2 diabetes mellitus is associated with accelerated muscle loss during aging, decreased muscle function, and increased disability [22]. As a weekly preparation of GLP-1RA, dulaglutide has been beneficial in improving the condition of diabetic patients with chronic kidney disease and cardiovascular disease in clinical trials [18, 19]. Some studies have found that GLP-1RA may affect skeletal muscles by affecting the expression of myokines. The GLP-1RA PF1801 can improve muscle weakness in polymyositis and suppress muscle inflammation by inhibiting muscle fiber necroptosis [17]. Animal studies have shown that dulaglutide improves muscle mass and strength, inhibits muscle atrophy factor, and increases the expression of the myogenic factor MyoD in aged mice, suggesting that dulaglutide may play a beneficial role in the treatment of muscle atrophy [23]. Fibronectin III domain containing 5 (FNDC5) is a type I transmembrane glycoprotein, the proteolysis of which at the carboxyl terminus releases irisin, which mainly comprises the fibronectin III domain of FNDC5 [24]. A meta-analysis found a direct positive correlation between serum-circulating irisin and insulin resistance in non-diabetic adults [25]. Through the assessment of body weight, body fat, and endothelial cell dysfunction markers, one study found that the increased plasma irisin level in patients with type 2 diabetes was correlated with the levels of obesity indicators, and irisin may be involved in atherosclerotic endothelial injury accompanied by obesity and type 2 diabetes mellitus [26].

Recent studies have shown that the myokine irisin affects bone metabolism *in vivo*. Mice treated with irisin showed improvements in cortical bone mass, geometry, and strength, similar to how physical activity affects the development of adequate weight-bearing bone. Irisin is a potential biomarker of muscle dysfunction that can help predict the onset of sarcopenia and provide a new way to monitor age-related muscle changes [27].

One study investigated the effect of the GLP-1RA exenatide on irisin levels in newly diagnosed obese patients with T2DM. Changes in the irisin level after treatment with exenatide were associated with decreases in FBG and HbA1c levels. Upregulation of irisin may be a new mechanism underlying the effect of exenatide in the treatment of type 2 diabetes mellitus [28]. Nother study explored changes in serum irisin and IL-6 levels in patients with T2DM after 6 and 12 months of treatment with a GLP-1RA. Further treatment with GLP-1 analogues increased the serum-circulating irisin level and decreased the IL-6 level. Changes in the irisin level after treatment with GLP-1 were associated with a decrease in total cholesterol, while changes in the IL-6 level were associated with a reduction in the waist circumference [29]. In the present study, the level of serum-circulating irisin in patients with diabetes mellitus was reduced, consistent with previous studies.

It has been reported that a high Neutrophil/Lymphocyte Ratio (NLR) is associated with the risk of sarcopenia in hospitalized patients with cancer [30, 31]. A multicenter prospective longitudinal sarcopenia study was conducted in the Peking Union Medical College Hospital and involved 56 elderly patients with sarcopenia and 56 elderly non-sarcopenia patients. The study found that, compared with those in non-sarcopenia patients, the serum levels of IL-6, IL-18, TNF-*α*, TNF-like weak inducing factor of apoptosis (TWEAK), and leptin were significantly increased in sarcopenia patients [32]. In the skeletal muscle of TNF-*α*-overexpressing transgenic mice, TNF-*α* levels were increased, accompanied by muscle atrophy, reduced numbers of satellite cells and type IIa muscle fibers, and an increased number of myeloid cells, including macrophages and granulocytes. An increased expression of TNF-*α* in myeloid cells impairs their differentiation [31].

Overall, in the present study, we examined the expression levels of the inflammatory cytokines IL-1*β*, IL-6, and TNF-*α* and the inflammatory chemokines CCL2, CXCL1, and CXCL2 in the muscle tissues of aged db/db mice before and after dulaglutide intervention. The results showed that the levels of CCL2, CXCL1, CXCL2, IL-1*β*, and IL-6 in the muscle tissues of db/db mice were significantly increased. The increase in these levels was not significant, which may be related to individual differences. Dulaglutide could significantly reduce these increased levels of IL-1*β*, IL-6, CCL2, and CXCL1. Increased levels of chemokines can recruit a large number of inflammatory cells, such as macrophages and neutrophils, to aggravate muscle tissue damage, and dulaglutide can reverse this inflammatory cell infiltration effect, suggesting that dulaglutide can reduce muscle tissue injury in aged diabetic mice, partly by inhibiting inflammation. This is similar to our previous study [33].

The present study has the following limitations. First, the treatment effects of dulaglutide on diabetic sarcopenia patients is unknown and need further investigation. Second, due to the irisin release caused by FNDC5, the level of irisin under dulaglutide treatment should be determined in order to research the relationship between myokines and diabetic sarcopenia in depth. In summary, our present study showed the protection effect of dulaglutide against muscle tissue injury in mice with diabetic sarcopenia by inhibiting inflammation and regulating the differentiation of myoblasts. In addition, we hope that our study can provide new therapeutic ideas for the targets of diabetic sarcopenia in the future.

## Data Availability

All the data generated or analyzed during this study are included in this article. Further enquiries can be directed to the corresponding author.

## Supplementary Materials

Supplementary Materials Supplementary Figure 1 C2C12 cells differentiate into skeletal muscle cells upon stimulation with horse serum. A The differentiation of C2C12 cells was induced by 2% horse serum, and the morphological changes of myocytes were observed under a microscope with a scale of 100 *μ*m. B-C RNA was extracted from C2C12 cells after differentiation, and the mRNA expression levels of differentiation genes MyHC4 and MyHC7 and myokine factors MyoD and MyoG were detected. ^*∗*^*P* < 0.05, ^*∗∗*^*P* < 0.01, ^*∗*^indicates the comparison of 2% horse serum group with CON group. Supplementary Table 1. The primer sequences used for qPCR.
