# Supplementary material for: Dulaglutide Protects Mice against Diabetic Sarcopenia-Mediated Muscle Injury by Inhibiting Inflammation and Regulating the Differentiation of Myoblasts
Source: Int J Endocrinol. 2023 Aug 7;2023:9926462. doi: 10.1155/2023/9926462 (PMC10425251; doi:10.1155/2023/9926462)
Supplement: Supplementary Materials — Supplementary Figure 1 C2C12 cells differentiate into skeletal muscle cells upon stimulation with horse serum. A The differentiation of C2C12 cells was induced by 2% horse serum, and the morphological changes of myocytes were observed under a microscope with a scale of 100 μm. B-C RNA was extracted from C2C12 cells after differentiation, and the mRNA expression levels of differentiation genes MyHC4 and MyHC7 and myokine factors MyoD and MyoG were detected. ∗P < 0.05, ∗∗P < 0.01, ∗indicates the comparison of 2% horse serum group with CON group. Supplementary Table 1. The primer sequences used for qPCR. [file 9926462.f1.docx]

**Supplementary figure and figure legend**


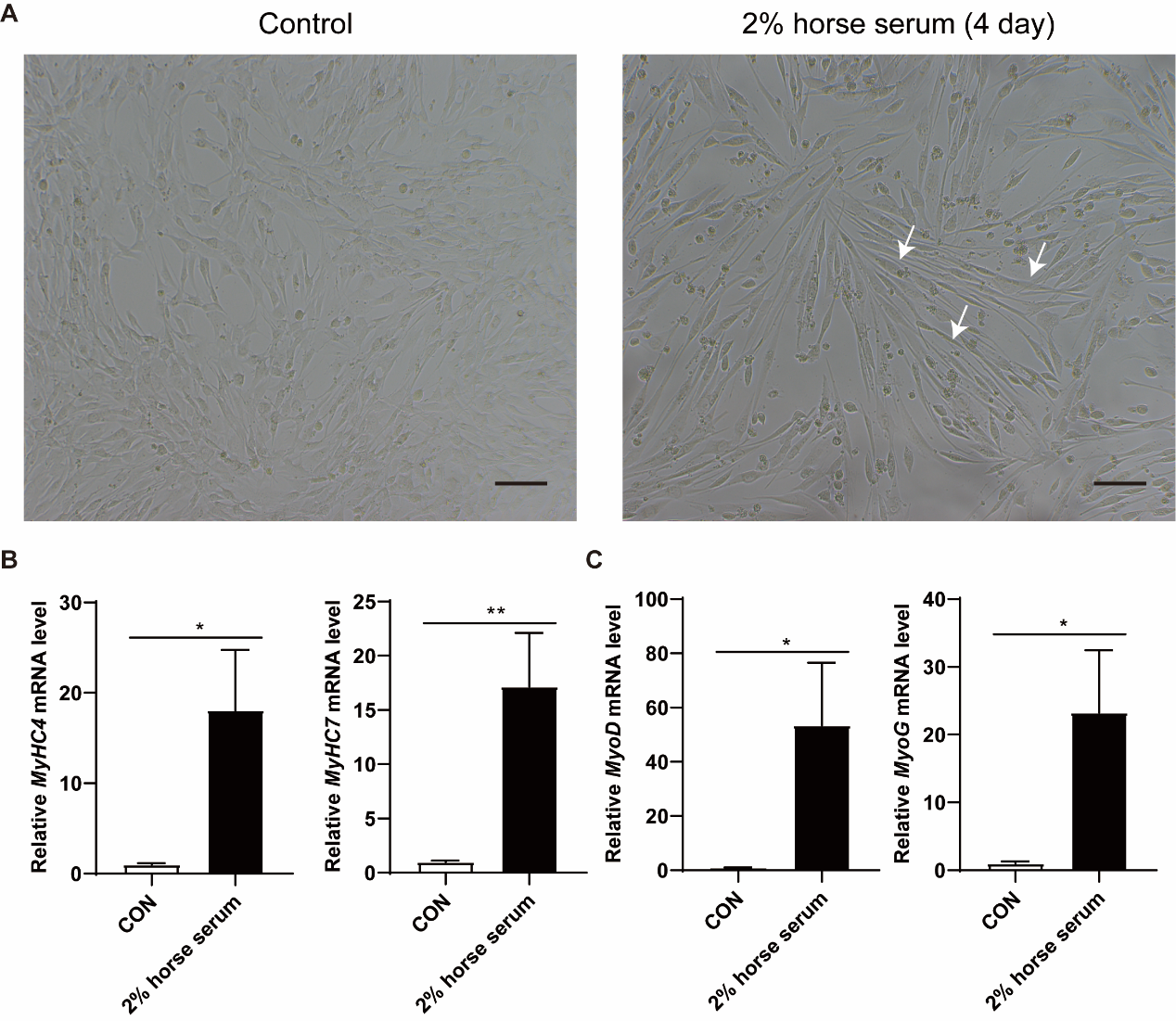


**Sup Fig. 1** C2C12 cells differentiate into skeletal muscle cells upon stimulation with horse serum. **A** The differentiation of C2C12 cells was induced by 2% horse serum, and the morphological changes of myocytes were observed under a microscope with a scale of 100 μm. **B-C** RNA was extracted from C2C12 cells after differentiation, and the mRNA expression levels of differentiation genes MyHC4 and MyHC7 and myokine factors MyoD and MyoG were detected. **P*<0.05, ***P*<0.01, *indicates the comparison of 2% horse serum group with CON group.

**Supplementary Table1**. The primer sequences used for qPCR.

| Gene | | Sequence (5’to 3’) |
| --- | --- | --- |
| mIL-1β | F | GGACAGAATATCAACCAACAA |
|  | R | TTACACAGGACAGGTATAGATT |
| mIL-6 | F | TAGTCCTTCCTACCCCAATTTCC |
|  | R | TTGGTCCTTAGCCACTCCTTC |
| mTNF-α | F | CCTGTAGCCCACGTCGTAG |
|  | R | GGGAGTAGACAAGGTACAACCC |
| mCcl2 | F | TTAAAAACCTGGATCGGAACCAA |
|  | R | GCATTAGCTTCAGATTTACGGGT |
| mCxcl1 | F | ACTGCACCCAAACCGAAGTC |
|  | R | TGGGGACACCTTTTAGCATCTT |
| mCxcl2 | F | CCAACCACCAGGCTACAGG |
|  | R | GCGTCACACTCAAGCTCTG |
| mMyHC4 | F | CAGACAGAGAGGAGCAGGAGAGTG |
|  | R | TTGGTGTTGATGAGGCTGGTGTTC |
| mMyHC7 | F | CAGAACACCAGCCTCATCAACCAG |
|  | R | TTCTCCTCTGCGTTCCTACACTCC |
| mFNDC5 | F | TGAACACCACCACCCGGTCC |
|  | R | GCCATCTTTTCAGCCTCGCG |
| mMyoD | F | CTTCTATCGCCGCCACTC |
|  | R | AAGTCGTCTGCTGTCTCAA |
| mMyoG | F | CCAACCCAGGAGATCATTTG |
|  | R | ACGATGGACGTAAGGGAGTG |
| mBDNF | F | TGCAGGGGCATAGACAAAAGG |
|  | R | CTTATGAATCGCCAGCCAATTCTC |
| mGAPDH | F | AGTGGCAAAGTGGAGATT |
|  | R | GTGGAGTCATACTGGAACA |
